# Pediatric trainees systematically under-report duty hour violations compared to electronic health record defined shifts

**DOI:** 10.1371/journal.pone.0226493

**Published:** 2019-12-12

**Authors:** Adam C. Dziorny, Evan W. Orenstein, Robert B. Lindell, Nicole A. Hames, Nicole Washington, Bimal Desai

**Affiliations:** 1 Department of Biomedical and Health Informatics, Children’s Hospital of Philadelphia, Philadelphia, Pennsylvania, United States of America; 2 Division of Critical Care Medicine, Department of Anesthesiology and Critical Care, Children’s Hospital of Philadelphia, University of Pennsylvania Perelman School of Medicine, Philadelphia, Pennsylvania, United States of America; 3 Department of Pediatrics, Emory University School of Medicine and Division of Hospital Medicine, Children’s Healthcare of Atlanta, Atlanta, Georgia, United States of America; 4 Division of General Pediatrics, Department of Pediatrics, Children’s Hospital of Philadelphia, University of Pennsylvania Perelman School of Medicine, Philadelphia, Pennsylvania, United States of America; Dalhousie University, CANADA

## Abstract

Duty hour monitoring is required in accredited training programs, however trainee self-reporting is onerous and vulnerable to bias. The objectives of this study were to use an automated, validated algorithm to measure duty hour violations of pediatric trainees over a full academic year and compare to self-reported violations. Duty hour violations calculated from electronic health record (EHR) logs varied significantly by trainee role and rotation. Block-by-block differences show 36.8% (222/603) of resident-blocks with more EHR-defined violations (EDV) compared to self-reported violations (SRV), demonstrating systematic under-reporting of duty hour violations. Automated duty hour tracking could provide real-time, objective assessment of the trainee work environment, allowing program directors and accrediting organizations to design and test interventions focused on improving educational quality.

## Introduction

To reduce errors associated with fatigue, the Accreditation Council for Graduate Medical Education mandates duty hour restrictions on trainees [1] but does not stipulate how monitoring should be accomplished [2]. Self-report systems are common [3] but are onerous for trainees to complete and vulnerable to recall bias and both under- and over-reporting [4,5]. Numerous single- and multi-institution surveys have demonstrated that trainees across many specialties substantially under-report duty hours [6–9]. Known reasons for under-reporting include the risk of loss of program accreditation [9], time for completion of electronic health record (EHR) documentation, guilt for leaving sick patients, and a “feeling that it was expected of them” [10,11]. However, the frequency of duty hour violations has not been measured objectively across a large sample of trainee shifts and rotations.

Trainees spend a substantial amount of time interacting with the electronic health record (EHR) [12,13]. Access logs store timestamps when users perform specific actions in the EHR. These logs provide objective measures of note-writing time [14], order entry workflow [15], time spent charting after hours [16] as well as trainee EHR usage [17]. Gilleland et al [16] suggested that adding objective EHR data to trainee self-reported duty hours would add an average of 1.44 new duty hour violations per trainee per month. Shine et al [18] found that average reported hours were 7.3% higher from self-reporting compared to EHR logs among internal medicine trainees on a single rotation, the medical intensive care unit.

We compared EHR-defined violations (EDV) to self-reported violations (SRV) across all inpatient rotations in a large pediatric residency program using an automated, validated algorithm to accurately identify provider shifts from electronic health record (EHR) logs [19].

## Methods

We included all pediatric trainees practicing on inpatient rotations at the Children’s Hospital of Philadelphia, a large academic training program in a quaternary care center, from 7/1/2015 through 6/30/2016. Trainees were classified as junior (e.g. interns at PGY-1) or senior trainees (e.g. PGY 2–4) in a front-line ordering clinician role (Jr FLC or Sr FLC), or senior trainees in a supervisory role (Sr Suprv). A resident-block was defined as a trainee working on a given rotation during a 4-week block. All blocks were 28 days in duration except for the first and last blocks of the academic year, which each contained an extra day (29 days). The number of scheduled shifts per block varied from approximately 17 with an every-fourth-night call schedule to 23 with a day-night shift schedule. Most resident-blocks included outpatient clinic one afternoon per week.

Log entries from the hospital EHR (Epic Systems^©^) were converted into EHR-defined shifts (EDS) using a validated algorithm [19]. Briefly, we extracted all access log event timestamps recorded for eligible trainees while excluding event timestamps from workstations not located in the hospital. We calculated event intervals as the difference between subsequent timestamps. We then defined EHR shifts by setting the starting timestamp after a “long” EHR event interval and the end timestamp prior to the next “long” EHR event interval. The “long” duration heuristic and subsequent shift refinement were defined based on our prior published validation process.

Self-reported shifts (SRS) were exported from New Innovations^©^ Resident Management Suite, a web-based self-reporting system which has been implemented in our training program for >3 years. Trainees were expected to log all clinical shifts in this system, with email reminders sent from training program administrators at regular intervals. We exported SRS for all included trainees several months following the completion of the academic year, allowing trainees sufficient time to complete duty hour logs. As this study was completed retrospectively, we did not engage specific trainees to ensure logging of all shifts but rather analyzed only the data present at the time of extraction.

Duty hour violations were computed for all shifts (EDS and SRS) across all resident-blocks. “Duration” violations were defined as trainee shifts exceeding maximum allowed shift duration, as defined at the time of this study (16 hours for interns; 24+4 hours for senior trainees). “Interval” violations were defined as inadequate time-off between shifts (<8 hours following shifts ≤16 hours; <14 hours following shifts >16 hours). We measured the number of violations by type across trainee roles for all EDS and calculated the block-by-block difference of total SRV and EDV for all resident-blocks with any SRS present. Continuous variables with multiple groups were analyzed with ANOVA followed by Tukey post-hoc testing to adjust for multiple comparisons. A multivariable regression model estimated the influence of predictors including role and source (‘EDV’ versus ‘SRV’) on the outcome of ‘total violations’. Regression coefficients are reported for each predictor or category of predictor. All analysis was completed in R Studio [20]. Computation was facilitated with the ‘dplyr’ package [21] and graphics were generated with ‘ggplot2’ and ‘cowplot’ packages [22,23].

This study was approved by the Institutional Review Board at Children’s Hospital of Philadelphia with a waiver of consent as trainee data were analyzed in aggregate.

## Results

Our study cohort included 139 trainees working 771 resident-blocks. Within these resident-blocks, trainee post-graduate years were represented as: 339 (44.0%) from PGY-1 trainees, 327 (42.4%) from PGY-2 trainees, 102 (13.2%) from PGY-3 trainees and 3 (0.4%) from PGY-4 trainees. Similarly, trainee roles were represented as: 339 (44.0%) as Jr FLC, 305 (39.6%) as Sr FLC and 127 (16.5%) as Sr Suprv. A total of 168 resident-blocks (22%) from 23 trainees (17%) contained zero self-reported shifts (SRS) and were excluded when the analyses included SRS. This exclusion resulted in 603 resident-blocks (78%) from 116 trainees (83%) eligible for paired EDV-SRV analysis.

**Fig 1:**
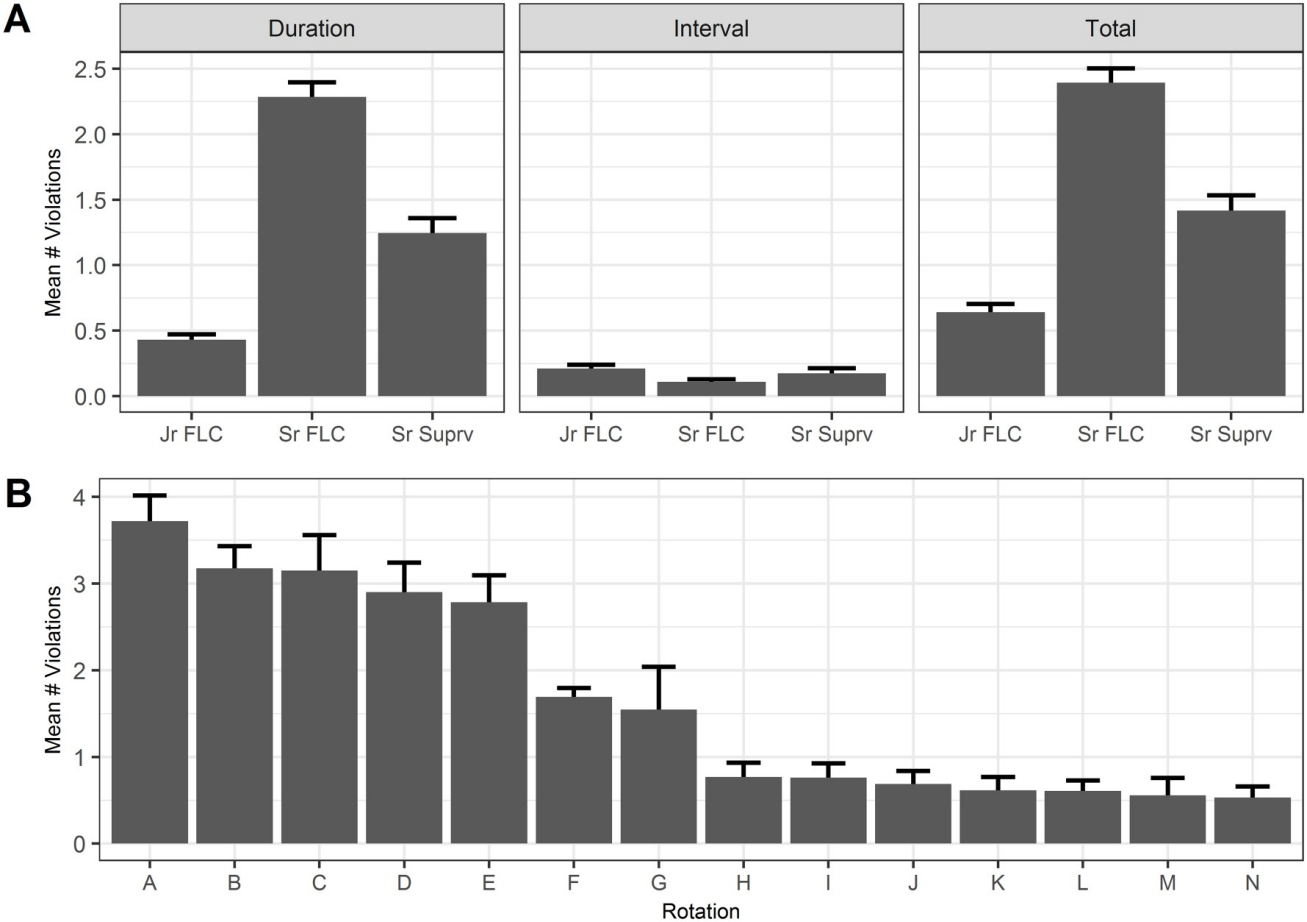
EHR-defined violations (EDV) vary by role and rotation. A, Mean number of duration, interval and total violations by trainee role. B, Mean number of violations by rotation. This figure includes all EDVs from all resident-blocks (N = 771).

Mean total EHR-defined violations (EDV) per block for all resident-blocks varied by role (Mean±SEM: Jr FLC 0.6±0.06, Sr FLC 2.4±0.11, Sr Suprv 1.4±0.12; *p* <0.001) [Fig 1A]. Across all EHR-defined ‘duration’ violations, median [IQR] shift length beyond maximum allowed shift duration was 36.3 minutes [16.4, 70.8]. Counts of violations varied significantly by rotation for front-line clinician roles (Jr FLC and Sr FLC), with mean violation count ranging from 0.5±0.1 to 3.7±0.3 per resident-block [Fig 1B].

**Fig 2:**
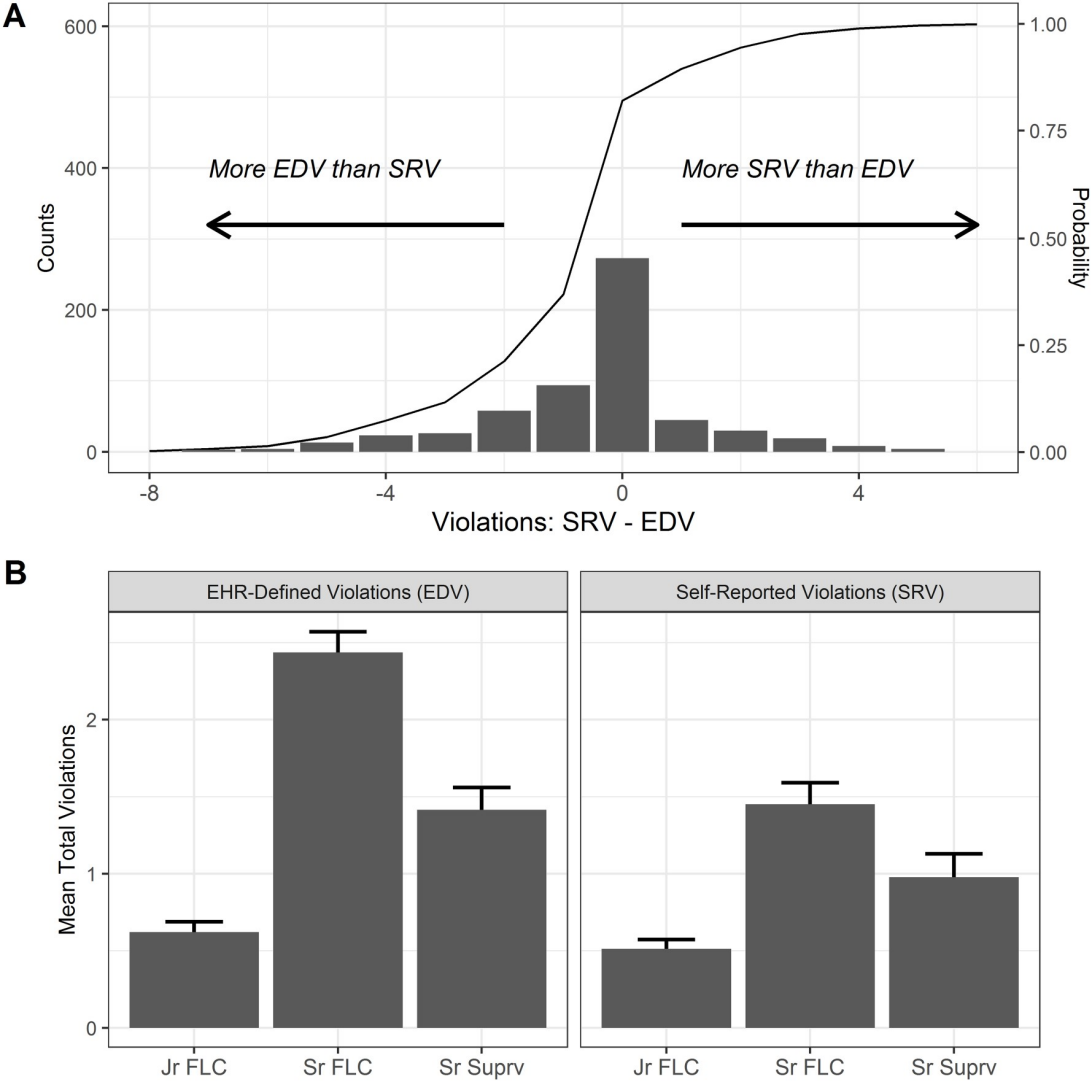
EHR-defined violations (EDV) exceed self-reported violations (SRV). A, Histogram of SRV minus EDV, where values less than zero indicate more EDV than SRV for an individual resident-block. B, Mean total violations by trainee role, grouped by EDV and SRV. SRV, Self-reported violations; ERV, EHR-reported violations. This figure only includes resident-blocks which included any SRV (N = 603).

In multivariable regression analysis, there were 0.45±0.09 fewer SRV than EDV (*p* <0.001) per resident-block, adjusted for trainee role. Additionally, there were greater total violations among trainees in the Sr FLC role (1.38±0.1) and Sr Suprv role (0.63±0.13) compared to Jr FLC (both *p* <0.001). Violations plotted as SRV-EDV show 36.8% (222/603) of resident-blocks with more EDV than SRV, while 17.9% (108/603) of resident-blocks had fewer EDV than SRV [Fig 2A]. This difference in EDV and SRV was most pronounced for the Sr FLC role. Mean total self-reported violations (SRV) per block also varied by role (Mean±SEM: Jr FLC 0.5±0.06, Sr FLC 1.5±0.14, Sr Suprv 1.0±0.15; *p*<0.001) [Fig 2B].

## Discussion

Automated ascertainment of trainee work hours using EHR logs demonstrated under-reporting of duty hour violations. To our knowledge, this is the first study to objectively examine a full academic year of trainees’ EHR- and self-reported shifts. Tracking duty hours in an automated fashion has several advantages to self-report including less onerous work for the trainee, avoiding recall bias [4,5], and limiting trainee’s disincentive to accurately report duty hours to avoid their program being placed on probation status [8]. Similar objective duty time systems are employed in the aviation industry where alertness on duty is critical, as part of Fatigue Risk Management Systems [24]. Additionally, prospective studies comparing different residency program duty-hour policies would benefit from such objective measuring of actual hours worked instead of relying on trainee reporting [25].

In this study we found 36.8% of resident-blocks had fewer SRV than EDV, consistent with survey-based studies suggesting 18–72% under-reporting [4,8,9]. Duration violations are the more frequent type of violation among all trainees, and interval violations trend towards being more common among Jr FLCs. Interval violations occur approximately once every 5 trainee blocks for Jr FLCs, and only once every 10 resident-blocks for Sr FLCs. However duration violations occur on average more than twice per resident-block among Sr FLCs, and more than once per resident-block among Sr Suprv trainees. On some rotations duration violations occurred nearly once per week, a substantial burden which can contribute to worse clinical performance [26] and increased medical errors [27].

This study was limited to a single institution and does not include out-of-hospital EHR logs by design. Because actual shifts may exceed the boundary of in-hospital EHR use, this method may under-estimate but should not over-estimate duty hours for inpatient shifts. Accordingly, we observed 18% of resident-blocks with more SRV than EDV. This may represent either over-reporting of duty hour violations using self-reporting tools, or actual shift duration exceeding the bounds of EHR use. Additionally, 22% of resident-blocks were excluded from paired analysis because no self-reported shifts were logged by trainees.

Automated duty hour tracking could provide real-time, objective assessment of the trainee work environment. Such systems would allow program directors to design and test interventions focused on improving the medical educational experience, quickly targeting rotations with more violations while simultaneously decreasing trainee non-clinical clerical tasks.
